# Dominant Influence of Interface Roughness Scattering on the Performance of GaN Terahertz Quantum Cascade Lasers

**DOI:** 10.1186/s11671-019-3043-6

**Published:** 2019-06-17

**Authors:** Junyan Cheng, Patrick Quach, Ding Wang, Fang Liu, Shangfeng Liu, Liuyun Yang, Huapeng Liu, Bo Shen, Yuzhen Tong, Xinqiang Wang

**Affiliations:** 1 State Key Laboratory of Artificial Microstructure and Mesoscopic Physics, School of Physics, Peking University, Beijing, 100871 China; 2 Collaborative Innovation Center of Quantum Matter, Beijing, 100871 China; 3 Nano-optoelectronics Frontier Center of Ministry of Education (NFC-MOE), Peking University, Beijing, 100871 China

**Keywords:** Quantum cascade lasers, GaN, Terahertz, Interface roughness scattering

## Abstract

Effect of interface roughness of quantum wells, non-intentional doping, and alloy disorder on performance of GaN-based terahertz quantum cascade lasers (QCL) has been investigated by the formalism of nonequilibrium Green’s functions. It was found that influence of alloy disorder on optical gain is negligible and non-intentional doping should stay below a reasonable concentration of 10^17^ cm^−3^ in order to prevent electron-impurities scattering degradation and free carrier absorption. More importantly, interface roughness scattering is found the dominating factor in optical gain degradation. Therefore, its precise control during the fabrication is critical. Finally, a gain of 60 cm^−1^ can be obtained at 300 K, showing the possibility of fabricating room temperature GaN Terahertz QCL.

## Introduction

Terahertz (THz) spectral region is a subject of intensive research because of its potential applications in quality and security control, medical diagnosis, and telecommunication. However, its development has been hindered by the lack of available compact devices. Quantum cascade laser (QCL) is a promising candidate for developing powerful THz solid state sources [1, 2]. Up until now, the best THz QCL is based on GaAs, whose maximum operating temperature is about 200 K due to the low LO-phonon energy (36 meV) of GaAs [3, 4]. With the assistance of a magnetic field, this temperature can be raised up to 225 K. However, this method is unsuitable for wide-spread applications [5, 6]. When the temperature increases, electrons in the upper-level state can acquire enough thermal energy for activating non-radiative relaxations via LO-phonon emission towards the lower level state, hence destroying the population inversion. In comparison to GaAs, GaN has much higher LO-phonon energy (92 meV) and thus provides the possibility of producing THz QCL operating at room temperature [7–9]. Furthermore, GaAs-based QCLs cannot be operated in the 4.6–12 THz frequency range because of their Reststrahlen band, the spectral region where the material is completely opaque due to the absorption by optical phonons. The larger energy of optical phonons in GaN opens prospects for THz quantum cascade devices, which can operate in a much broader spectral range between 1 and 15 THz.

The first step study in GaN THz QCLs was the tuning of the intersubband (ISB) transition to the far-infrared domain. ISB absorption at THz frequencies has been observed in polar [10, 11] and nonpolar nitride-based quantum wells (QWs) [12–17]. THz operating ISB GaN-based detectors were demonstrated at 13 THz [18] and 10 THz [19], respectively. No electroluminescence demonstration in this range has been achieved so far, except for some controversial report from Hirayama group on the spontaneous electroluminescence from a QCL structure [20, 21]. Several theoretical studies have been published [7, 9, 22–26], among them, some investigate limiting factors of GaN THz QCL performances such as gain spectrum broadening due to very strong interactions between electrons and LO phonons in GaN [23].

In this article, we propose to complete these studies by analyzing other factors that can damage THz GaN QCL optical gain such as interface roughness of quantum wells, non-intentional doping, and alloy disorder. It was found that influence of alloy disorder on optical gain is negligible, and non-intentional doping should stay below a reasonable concentration of 10^17^ cm^−3^ in order to prevent electron-impurities scattering degradation and free carrier absorption [27]. Finally, we found that interface roughness scattering is the dominating factor in optical gain degradation. And a gain of 60 cm^−1^ can be obtained at 300 K, which is well above the theoretical loss of a double metal waveguide, showing the possibility of fabricating room temperature GaN THz QCL.

## Methods

**Fig. 1:**
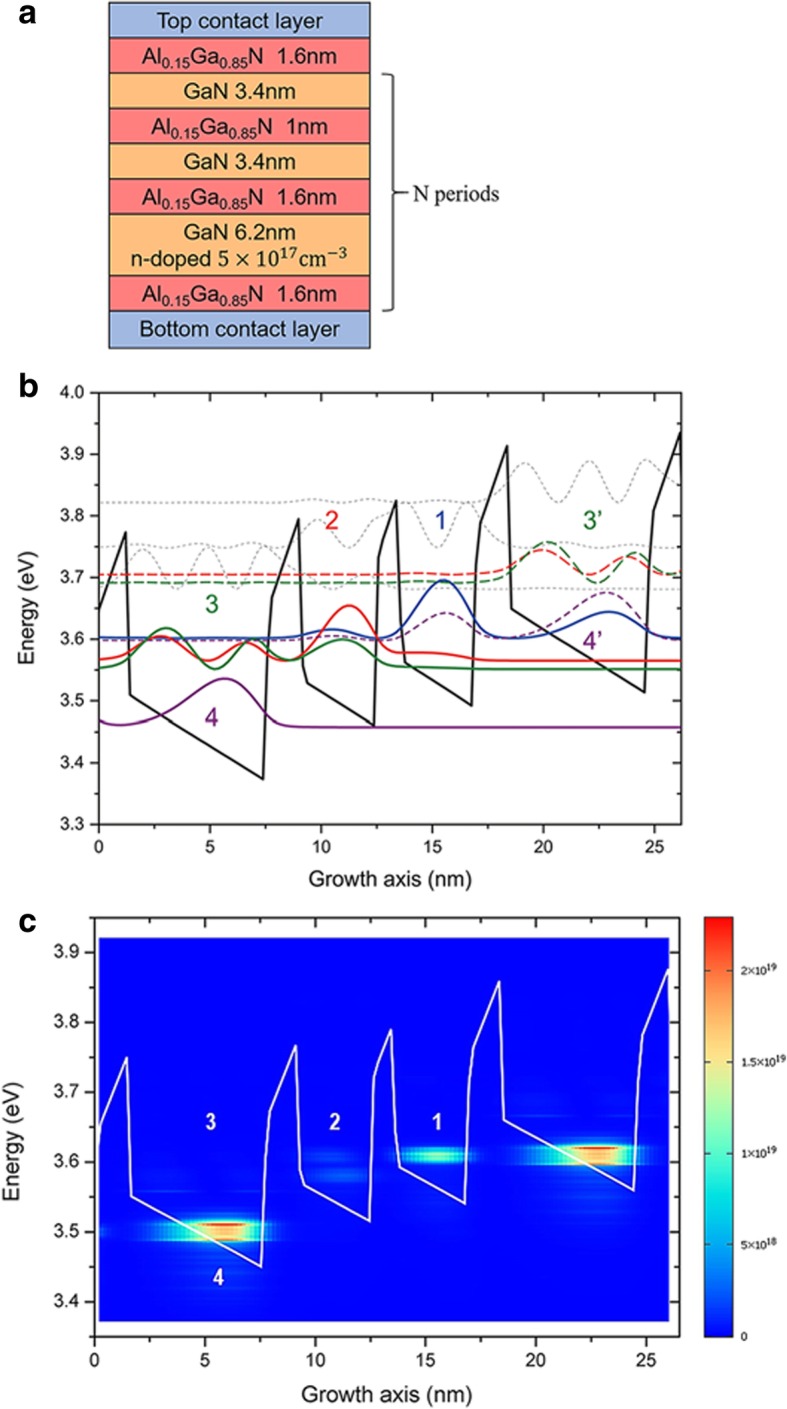
Designed active region structure, conduction band profile, squared envelope functions, and carrier densities. **a** The layer sequence for one period is *1.6*/6.2/*1.6*/3.4/*1.0*/3.4 nm. Barriers are indicated in italics font. The 6.2 nm-thick well is n-doped with *n* = 5 × 10^17^ cm^−3^. **b** Conduction band profile and squared envelope functions of the GaN/Al_0.15_Ga_0.85_N QCL considered in this study. An electric field of − 84.5 kV/cm is applied. **c** Carrier densities and conduction band of the QCL calculated in the NEGF model. The electric field applied is − 84.5 kV/cm. Temperature is set at 10 K.

It is known that fabrication of GaN THz QCL devices needs to grow thick active regions with low dislocation densities. This task is challenging because of the lattice mismatch between GaN and AlGaN [28]. Other unwanted factors coming from epitaxy can also appear: interface roughness (IFR) depending on growth condition, n-type non-intentional doping (n.i.d) coming from impurities (mostly oxygen) incorporation during growth and alloy disorder (AD) originating from Ga surface segregation and Al adatom low mobility. To investigate how these phenomena influence THz GaN QCL performance, we use the formalism of nonequilibrium Green’s functions (NEGF). Calculations are performed using Nextnano QCL software [29–31]. This model includes relaxation induced by interface roughness, ionized impurities, alloy disorder, LO phonon, acoustic phonon, or electron-electron interaction. We employed a three-quantum-well QCL with a resonant-phonon depopulation scheme since that THz QCL design provides the highest operating temperature till now [3, 32]. Figure 1a shows the designed active region structure. The layer sequence for one AlGaN quantum structure/AlGaN quantum structure is *1.6*/6.2/*1.6*/3.4/*1.0*/3.4 nm, where the italics ones show thickness of barriers. Figure 1b shows the conduction band diagram of the designed QCL structure at a bias of − 84.5 kV/cm. From the previous period on the right, electrons are injected by resonant tunneling in the upper lasing state, labeled by 1. Then, they undergo a radiative transition to the lower lasing state 2. This lower lasing state is depopulated through tunneling to state 3. Finally, electrons relax through into state 4 by LO-phonon emission. The process is repeated for each period.

In the calculation, we use the parameters usually found in GaN/AlGaN grown structure with plasma-assisted molecular beam epitaxy (PAMBE): an interface roughness of 0.25 nm [33] with a correlation length of 1 nm and a non-intentionally n-doping with a carrier concentration of 10^17^ cm^−3^. Alloy disorder scattering is also included in the simulation.

## Results and Discussion

**Fig. 2:**
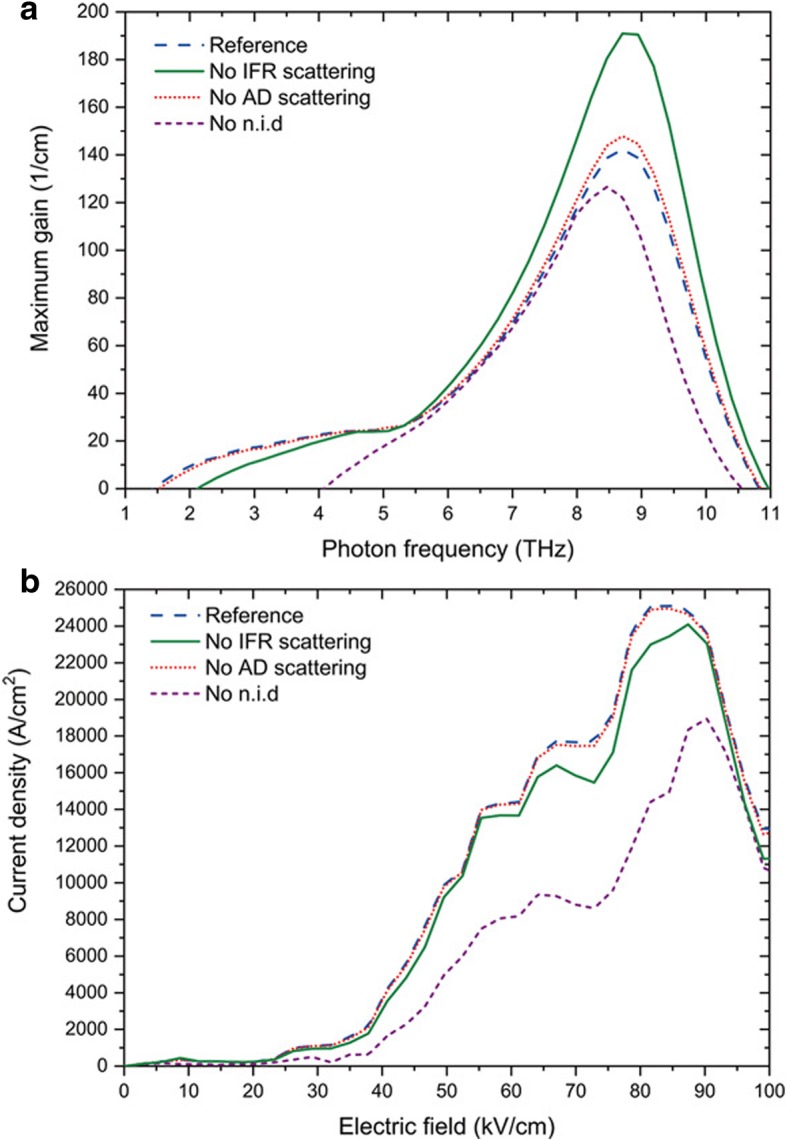
Simulated maximum optical gain vs frequency and current-electric field simulations for different scattering process. **a** Simulated optical gain vs frequency taking account of different scattering process. **b** Current-electric field simulations of the GaN THz QCL taking account different scattering parameters. Temperature is set at 10 K

Figure 1c shows the calculated carrier densities of this structure at the operating bias of − 84.5 kV/cm. We observed the anti-crossing between the previous period and the upper lasing state 1. We also see that the lower lasing state 2 is depopulated by resonant phonon in state 3 and 4. In order to analyze and compare the influence of IFR, n.i.d, and AD, we calculated our GaN THz QCL optical gain and current characteristics for several configurations: the reference configuration taking IFR, n.i.d, and AD into account, a configuration without IFR, another one without n.i.d, and a last one without AD. Figure 2 shows the maximum optical gain vs frequency (a) and current densities vs applied electric field (b) for each configuration calculated at a temperature of 10 K. The reference structure shows a peak gain of 142 cm^−1^ at 8.7 THz, frequency unreachable for arsenides material. Let us see how n.i.d influences our QCL performance. Without n.i.d, the peak gain is 127 cm^−1^ at 8.46 THz. The gain drop is due to that carrier concentration decreases in the upper lasing state after taking away electrons coming from n.i.d. Indeed, in the reference configuration, electron concentration of the upper and lower lasing state is *∆N* = *N*_1_ – *N*_2_ = 5.43 ×10^12^cm^−2^, while without n.i.d it becomes *∆N* = *N*_1_ – *N*_2_ = 5.06 ×10^12^cm^−2^. Applied electric field shifts from − 84.5 to − 81.6 kV/cm. Current threshold drops and shifts from 25.11 kA/cm^2^ at − 84.49 kV/cm to 17.11 kA/cm^2^ at − 93.24 kV/cm. Current density drop can be attributed to the reduction of electron-impurities scattering which increases electrons transport in the calculation without n.i.d. Another hint of this hypothesis is the peak at − 64 kV/cm that we see in the case without n.i.d current densities characteristics. This is an inter-period resonant tunnel between 4’ and 3 (not shown here). This phenomenon is hidden by electron-impurities scattering in the current characteristics taking account of n.i.d. The current threshold and applied electric field shift are attributed to conduction band misalignment between the configuration with or without n.i.d. Interestingly, even though the gain peak is larger in the n.i.d case, we observe a gain spectrum broadening, the signature of charged impurities influence [31] Non-intentional doping should stay at a reasonable concentration of 10^17^ cm^−3^ to prevent electron-impurities scattering degradation and free carrier absorption. In the configuration without AD scattering, peak gain is 147 cm^−1^ at 8.7 THz. We observe that peak gain is at the same frequency with or without AD scattering. Optical gain only gets a marginal increase of 3% when AD scattering is not included in the calculation. Current characteristics are also almost identical. Since our design uses a low aluminum content of 15% and fairly thin barriers (1–1.5 nm), AD scattering influence in this QCL is negligible.

On the contrary, IFR scattering influence in the device’s performance is important. Without IFR scattering, we observe a peak gain of 191 cm^−1^ at 8.7 THz. Current density threshold is 24.08 kA/cm^2^. This gain increase of 34% and the current density threshold drop reflects the fact that a lot of electrons are transported through IFR scattering. The more IFR scattering, the less radiative scattering there is for lasing. When comparing the reference configuration electrons population of the upper and lower lasing state *∆N* = *N*_1_ – *N*_2_ = 6.6 ×10^12^ – 1.27 ×10^12^ = 5.43 ×10^12^cm^−2^ to the one without IFR *∆N* = *N*_1_ – *N*_2_ = 7.4 ×10^12^ – 1.17 ×10^12^ = 6.23 ×10^12^cm^−2^, one can see that the upper state electrons population is higher. This is due to the upper lasing state lifetime which increases due to the absence of IFR scattering. In comparison to the case without n.i.d, in the current densities characteristics of the device without IFR scattering, we observe a peak at − 67 kV/cm, signature of the inter-period resonant tunnel between 4’ and 3. This phenomenon is more visible in the case without taking IFR scattering process taken into account. This is a proof of its predominance over resonant tunneling. With those observations, we highlight the predominance of IFR scattering influence in the performance of THz GaN QCL.

**Fig. 3:**
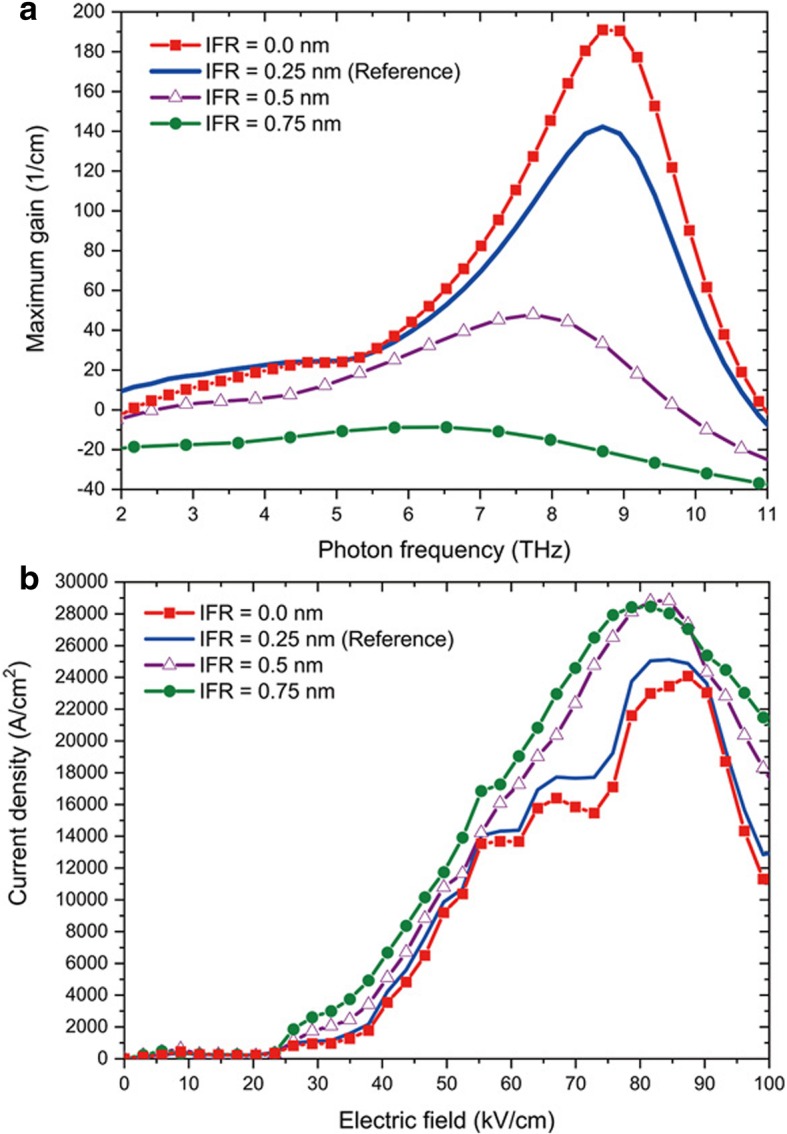
Simulated maximum optical gain vs frequency and current-electric field simulations for different IFR. **a** Simulated maximum optical gain vs frequency for different IFR. **b** Current-electric field simulations of the GaN THz QCL taking account different IFR. Temperature is set at 10 K

After noticing the importance of IFR scattering in THz performance. We investigated further by varying IFR size. We added to our study the case of IFR = 0.5 nm and 0.75 nm. Correlation length is kept at 1 nm. In figure 3, we showed the maximum gain vs frequency (a) and current densities vs applied electric field characteristics (b). First, we observed that for IFR = 0.5 nm, maximum optical gain decreases to 47.9 cm^−1^ and even dramatically drops to − 8.8 cm^−1^ losing optical gain for IFR = 0.75 nm. The gain broadening as a function of IFR length is also evident. As we can see in I-V characteristics, as IFR size increase, its role in electrons scattering increases, increasing current densities and diminishing resonant tunnel and radiative scattering process in the devices. This effect becomes evident when comparing the reference configuration of IFR = 0.25 nm to the extreme case of IFR = 0.75 nm, electrons population of the upper and lower lasing state dropping from *∆N* = 5.43 ×10^12^cm^−2^ to *∆N* = *N*_1_ – *N*_2_ = 3.71 ×10^12^cm^−2^.

The latter decreases to the point that we cannot see lasing in the devices anymore. As already pointed out in previous studies using GaAs-based THz QCL [25, 34–36], we highlight the importance of considering IFR size during epitaxy and of keeping it smaller than 0.5 nm for fabrication of GaN THz QCL to be able to provide positive optical gain.

**Fig. 4:**
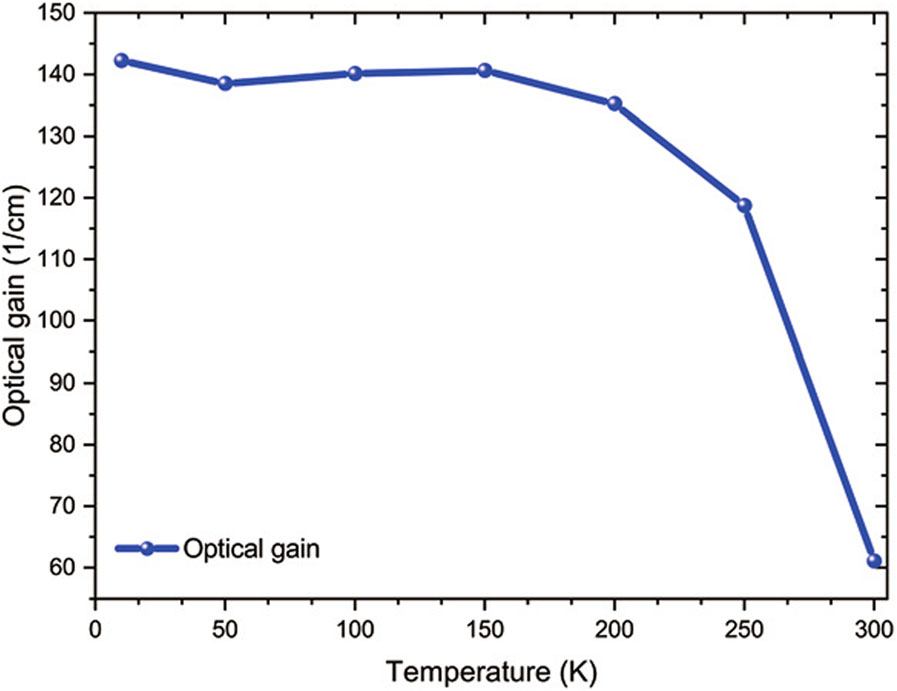
Characteristics of the calculated maximum gain vs lattice temperature

An advantage for GaN THz QCL is its potential to operate at a higher temperature than GaAs-based THz QCL. In this part, we analyzed our device performance as a function of operating temperature. We continued using our reference devices with IFR = 0.25 nm, n.i.d, and AD included in the calculation. Figure 4 shows the maximum optical gain for different lattice temperatures. The gain value is stable from 10 to 150 K at around 142 cm^−1^, it begins to decrease between 150 and 250 K, for dropping to 61 cm^−1^ at 300 K. Indeed, as temperature increases, population inversion decreases due to thermal backfilling and LO-phonon scattering increase induces gain broadening. This optical gain value of 61 cm^−1^ is still higher than the loss of a GaN THz QCL double metal waveguide (30 cm^−1^), showing that this GaN THz QCL design should be able to operate at room temperature. We also mention that besides being able to operate at room temperature, GaN-based THz QCL has another advantage: because of their higher doping concentration, lower refractive index, and thinner period length, they have the potential to provide much higher optical gain than in their GaAs counterpart. Our design provides a fairly high optical gain value of 142 cm^−1^ at 10 K, which is a good example.

## Conclusions

In conclusion, we report a GaN THz QCLs design operating at 8.7 THz. The simulation shows an optical gain of 142 cm^−1^ at 10 K and 60 cm^−1^ at room temperature. Among unwanted phenomena originating from epitaxy, we have calculated the influence of interface roughness, non-intentional doping, and alloy disorder in the performance of GaN THz QCL gain. Alloy disorder influence is neglectable: optical gain only drops from 147 to 142 cm^−1^ when adding alloy disorder scattering in the simulation. Non-intentional doping should be taken into account in the design to prevent conduction band misalignment. We did observe applied electric field shift from − 84.5 to − 81.6 kV/cm induced by n.i.d in our study. Finally, we observed a great disparity in optical gain for different interface roughness values: 191, 142, 47.9, and − 8.8 cm^−1^ for interface roughness equal to 0, 0.25, 0.5, and 0.75 nm, respectively. That is why we identify the dominant influence of interface roughness scattering in the degradation of optical gain. This work provides routes for performance optimization of eventually future GaN THz QCL fabrication.

## Data Availability

The datasets generated during and/or analyzed during the current study are available from the corresponding author on reasonable request.

## Abbreviations

AD: Alloy disorder
IFR: Interface roughness
ISB: Intersubband
n.i.d: Non-intentional doping
NEGF: Nonequilibrium Green’s functions
QCL: Quantum cascade laser
